# Influence of Social Determinants, Lifestyle, Emotional Well-Being and the Use of Unconventional Therapies in Breast Cancer Progression in a Cohort of Women in Barcelona: Protocol for the DAMA Cohort

**DOI:** 10.2196/resprot.7653

**Published:** 2017-12-18

**Authors:** Rosa Puigpinos-Riera, Xavier Continente, Gemma Serral, Xavi Bargalló, Montserrat Doménech, Martín Espinosa-Bravo, Jaume Grau, Francesc Macià, Rafael Manzanera, Margarida Pla, M Jesus Quintana, Maria Sala, Eulalia Vidal

**Affiliations:** ^1^ Servei d'Avaluació i Mètodes d'Intervenció Agència de Salut Pública de Barcelona Barcelona Spain; ^2^ Centro de Investigación Biomédica en Red de Epidemiologia y Salud Pública Madrid Spain; ^3^ Institut de Recerca Sant Pau Barcelona Spain; ^4^ Agència de Salut Pública de Barcelona Barcelona Spain; ^5^ Hospital Clínic Barcelona Spain; ^6^ Universitat de Barcelona Barcelona Spain; ^7^ Association of Women with Breast Cancer (Agata) Barcelona Spain; ^8^ Hospital Vall d'Hebron Barcelona Spain; ^9^ Parc de Salut Mar Barcelona Spain; ^10^ Institut Hospital del Mar d'Investigació Mèdica Barcelona Spain; ^11^ MC Mutual Barcelona Spain; ^12^ Hospital de la Santa Creu i Sant Pau Barcelona Spain; ^13^ Institut Municipal d'Investigacions Mèdiques Barcelona Spain; ^14^ Facultat de Ciències de la Salut Blanquerna Universitat Ramón Llull Barcelona Spain

**Keywords:** breast cancer, cohort study

## Abstract

**Background:**

Breast cancer continues to be the most commonly diagnosed cancer in women. Breast cancer survivors face numerous problems, especially after completing the first year of intense treatment. We present the protocol for an ongoing study to analyze the impact of a series of factors on breast cancer survival related to lifestyle, emotional well-being, and use of complementary and alternative medicine (CAM).

**Objective:**

We aim to analyze the influence of social determinants, lifestyle changes, emotional well-being, and use of CAM in the progression of breast cancer in women diagnosed with breast cancer between 2003 and 2013 in Barcelona, Spain.

**Methods:**

We will perform a mixed cohort study (prospective and retrospective) of women diagnosed with breast cancer, created using a convenience sample in which we study the evolution of the disease (relapse, death, or remaining disease-free). Once identified, we sent the women information about the study and an informed consent form that they are required to sign in order to participate; a total of 2235 women were recruited. We obtained the following information from all participants: sociodemographic profile via a phone interview, and a self-administered survey of information about the study’s objectives (lifestyles, emotional well-being, health care services, and the use of CAM). Lastly, we examined clinical records to obtain data on the tumor at the time of diagnosis, the treatment received, the occurrence of relapses (if any), and the tumor typology. We present data on the women’s social profile based on descriptive data obtained from the telephone interview (welcome survey).

**Results:**

Based on the welcome survey, which was completed by 2712 women, 14.42% (391/2712) of respondents were <50 years of age, 45.50% (1234/2712) were between 50 and 65 years of age, and 40.08% (1087/2712) were >65 years of age. A total of 43.69% (1185/2712) belonged to the highest social classes (I and II), 31.27% (848/2712) to the middle class (III), and 23.49% (637/2712) to the working classes (IV and V). Approximately 22.71% (616/2712) lived alone, 38.31% (1039/2712) lived with one person, and 38.97% (1057/2712) lived with two or more people.

**Conclusions:**

We obtained information from a large cohort of women, but this study has limitations related to the convenience sampling strategy, one of which is reduced representativeness. Conversely, being a self-administered survey, the study introduces biases, especially from respondents that answered on paper. However, the information that the study provides will serve as the basis for designing future interventions aimed at improving the knowledge gaps indicated for women with breast cancer.

## Introduction

Breast cancer continues to be the most commonly diagnosed cancer in women [1]. In 2012, there were more than 25,000 new cases in Spain; in Catalonia, 1 in 9 women will be diagnosed with breast cancer in her lifetime [2]. Due to population aging, early detection, and diagnostic improvements, this trend will increase and the number of new cases is likely to rise by 70% in the coming decades [1]. Diagnostic improvements and treatment have also increased survival; in Catalonia the mortality rate has declined by 2.1% each year since 1993, such that current survival at 5 years is 82.8% [2].

Breast cancer survivors face numerous problems, especially after completing the first year of intense treatment. These problems keep survivors closely tied to hospitals, and they become aware that the physical and emotional consequences not only continue, but sometimes worsen. Survivors experience physical changes [3] that result in changes in their intimate life [4,5] and employment [6], as well as fatigue and pain associated with the disease that is detrimental to their mental health and quality of life [7]. The environment and social networks of survivors are vital to their quality of life and survival, and recent studies have shown that the biological mechanisms triggered in socially isolated individuals increase their risk of poorer results in terms of survival [8].

Depression and anxiety are the mental illnesses that most commonly affect women, and signs of these diseases are observed in 20-30% of breast cancer patients within the first year after diagnosis; in comparison, the general population prevalence of depression and anxiety are just 8% and 6%, respectively, according to studies in Switzerland and the United States [9]. According to the *European Study of the Epidemiology of Mental Disorders - Spain* study carried out in 2001-2002, the prevalence/year (new and old cases cases) of depression and anxiety were 5.6% and 1.18%, respectively [10].

Lifestyles factors such as diet, physical activity, tobacco usage, and alcohol consumption are directly or indirectly related to breast cancer, and to the general health of any person. In this sense, one of the basic principles of the European Code Against Cancer [11] is to maintain a healthy life in terms of a balanced diet and physical activity, and to reduce or avoid toxic habits.

To cope with secondary side effects, improve quality of life, and feel better, people often resort to complementary and alternative medicines (CAMs). In the United States, an estimated 28-73% of breast cancer survivors use one or more of these therapies [12]. Such therapies are also used in in Spain [13], although to our knowledge there is currently no data on the frequency of use. Acupuncture is an effective treatment for nausea and pain, and recent studies in patients undergoing hormone treatment have demonstrated that these therapies reduce hot flashes, anxiety, depression, and somatic and vasomotor symptoms [14].

However, since these strategies are not included in cancer follow-up protocols, their use depends on each woman's own resources, which may lead to important inequalities. According to the Wisconsin Longitudinal Study, overall health status and the appearance of a particular breast cancer can be influenced not only by the status of the individual herself in the present moment of her life, but also by the history of her family [15]. Socioeconomic status and the environment both influence quality of life among women with breast cancer. Both of these factors are linked to the emotional stress caused by the disease and the difficulties added to job loss, lack of support in housework, lack of support in changing lifestyles, and lack of information and financial resources to adopt and maintain habits [16]. Some of these habits are a healthy diet that may involve dietitian visits, or access to certain types of foods, exercises, or therapies [17].

For this reason, it is important to analyze the needs and problems related to breast cancer patients’ lifestyles (tobacco and alcohol consumption, eating habits, physical exercise), emotional well-being (quality of life, social networks, mental health, relationship with health services, employment situation), and use of CAM, and how these factors can influence survival. Thus, we designed the *Dones Amb Càncer de Mama* (DAMA) Cohort of women diagnosed with breast cancer, and in this paper we present the protocol that was followed to gather information and construct the profile of the women in this cohort.

### Objectives

The main objective of the study is to analyze the influence of social determinants, lifestyle changes, emotional well-being, and use CAM in the progression of breast cancer in women diagnosed with breast cancer between 2003 and 2013 in Barcelona. Specific objectives include: (1) retrospectively describing lifestyles before and after breast cancer, according to socioeconomic status; (2) describing emotional well-being and use of CAM among women diagnosed with breast cancer, according to socioeconomic status; (3) describing patients’ clinical progression from the time of breast cancer diagnosis to the start of the study; (4) evaluating whether lifestyle, emotional well-being, and the use of CAM are associated with tumor progression, according to socioeconomic status; and (5) analyzing the influence of contextual determinants on breast cancer progression according to lifestyles, emotional well-being, and the use of CAM.

## Methods

### Study Population

We included women aged >18 years who were diagnosed with and/or treated for breast cancer in the four most important hospitals in the Barcelona Public Hospital Network (Hospital Clínic, Hospital Vall d’Hebrón, Hospital de Sant Pau, Parc de Salut Mar) between January 1, 2003 and December 31, 2013. Subjects were women from the Minimum Basic Data Set who, at the time of admission in the hospital, coded with any of the codes between 174.0 and 174.9 of the 9th revision of the International Classification of Diseases (ICD-9). Inclusion criteria were: (1) aged >18 years, (2) admitted to hospital with a main diagnosis of breast cancer, and (3) diagnosed or treated at any time during the study period. Exclusion criteria were: (1) died from any cause before the start of the study, (2) diagnosed with any other type of cancer before being diagnosed with breast cancer, and (3) living outside Catalonia, due to difficulties in follow-up.

### Study Design

We performed a mixed cohort study (prospective and retrospective) [18] using a convenience sample of women diagnosed with breast cancer. We studied disease progression (recurrence, death from breast cancer, death from a cause other than breast cancer, or remaining disease-free), and exposure factors (lifestyle, nutritional changes, quality of life, social network, emotional well-being, and use of CAM, among others).

### Recruitment

We recruited women diagnosed with and/or treated for cancer at different stages, with and without relapse between 2003 and 2013. Thus, the cohort consists of women diagnosed with breast cancer at different phases of disease progression, and with information from the time of diagnosis onward. Candidates for inclusion received a letter from the hospital inviting them to participate. This letter was accompanied by two further documents: (1) information about the study, explaining the objectives, the institutions and hospitals involved, and the women’s rights if they decided to participate in the study; and (2) two informed consent forms signed by the lead investigator of the study, one of which was a copy for the participant to keep, and the other to be mailed back in a postmarked envelope.

Coinciding with the beginning of the recruitment phase, we sent information to the participants and to professionals involved in their treatment and follow-up, to sensitize and inform them about the study. The professional recipients included: hospital specialists, primary care physicians, nurses, case managers, psycho-oncologists, and professionals in the Women's Sexual and Reproductive Health Care Program (PASSIR, in Spanish). We also distributed flyers with information about the study to hospitals, primary care centers, and Concerned Women’s Associations. Two months after sending the women this information for the first time, we resent it to those who had not yet responded.

### Ethical Approval and Consent to Participate

This study was evaluated first by the Clinical Research Ethics Committee of Parc de Salut Mar, and then by the corresponding committees of each hospital. Following the initial contact by the hospital, all women received two informed consent forms signed by the lead investigator; they kept one for themselves, and were asked to sign and return the other, which was an essential requirement for participation in the study. The informed consent form explained the subjects’ rights, including the right to withdraw from the study at any time. Completed surveys and the databases created using them were anonymized using a code to link all information exchanged between the hospital and the study coordinating center.

### Information Sources

We obtained information from various sources, including: (1) telephone interview and ad-hoc questionnaire; (2) hospital clinical records; (3) data from the National Statistics Institute, which we used to obtain ecologic indicators that then allowed us to analyze the influence of structural determinants; and (4) hospitals, relatives, and the Civil Register, which was our main source of the mortality information. The advantage of the civil register is that is contains almost real-time mortality data, while the mortality register provides data with almost two years delay. However, a disadvantage of the civil register is that it only records deaths that occur in Barcelona city, but those of women from Barcelona who died outside the city are not in this source of information.

### Field Work

We had three data collection points or phases: Initial Welcome Call, Study Survey, and *Review of Medical Records*.

#### Initial Welcome Call

After receiving the informed consent forms, we called each candidate by phone and administered the Welcome Survey, which was intended to accomplish three things: (1) welcome the participant to the study and thank her for her participation; (2) determine her social profile; and (3) explain how we would continue developing the study, proceed to the next phase, and continue the study survey itself. The study survey was self-administered, and participants had the option to complete it online or send it by post.

#### Study Survey

Using either conventional mail or email, we sent participants a link to complete the survey online. To help resolve doubts, we offered participants follow-up and telephone or mail support at all times. If individuals had not responded within a month after sending the survey, we sent them a reminder by email or post and resent the survey if necessary. This survey collected the following data: sociodemographic data, health data, data on breast cancer and treatment received, lifestyle (tobacco, alcohol, nutrition, physical activity), emotional well-being (including information about social networks, emotional support, quality of life, mental health), satisfaction with health services (health professionals, personal treatment, information received), and use of unconventional therapies. Table 1 shows the parts of the questionnaire in detail and the origin of the questions.

#### Review of Medical Records

**Figure 1:**
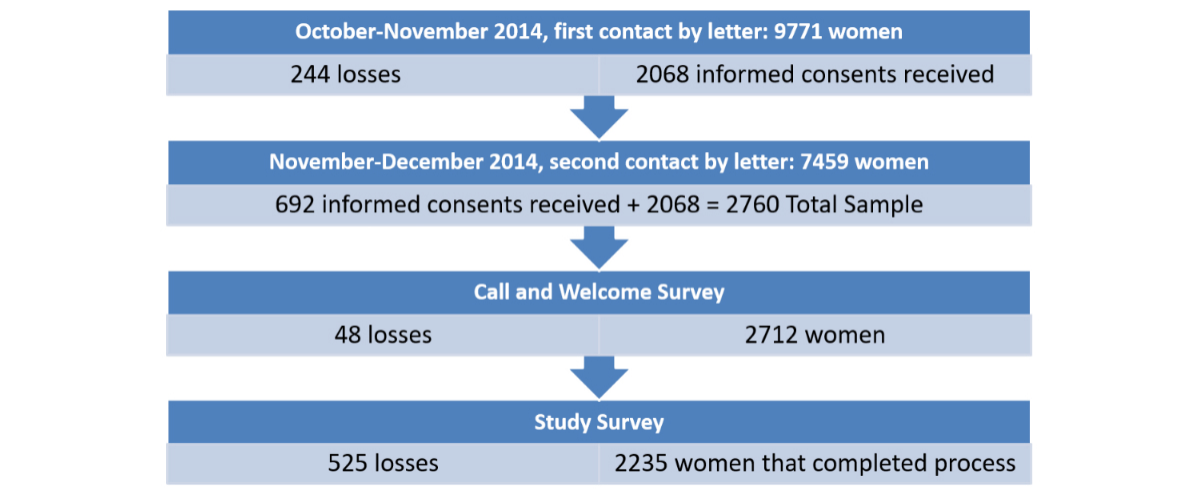
Overview of the study process.

After completing this process, we reviewed the participants’ clinical records, including: information at the time of diagnosis, tumor nodes metastases (TNMs; international classification of tumor, nodes, and metastases); status, biological characteristics, and characteristics of the tumor; treatment received; relapse; how the woman felt during her last visit to the hospital; and date. The number of women who followed the process from beginning to end can be seen in Figure 1.

### Study Variables

#### Dependent Variables

The main dependent variable is tumor progression or survival (relapse or metastasis, death, disease-free time). For objectives 1 and 2, the dependent variables are those related to: (1) lifestyle, including dietary habits, body mass index (BMI), smoking habits, and alcohol consumption (before and after diagnosis); (2) emotional well-being (quality of life, social networks, emotional support, and mental health), and use of CAM (type of CAM and frequency of use). Objective 3 is related to the clinical aspects of the tumor, and the dependent variables were: TNM at the time of diagnosis, stage, type of tumor (histology, hormonal receptors, Her2 expression), and type of treatment (mastectomy or not, reconstruction or not). For objectives 4 and 5, the dependent variable is tumor progression.

**Table 1:** Structure of the welcome survey and source of the questions.

Block and specific topic		Source
**Health data**		
	General health	ESB-2011 (Barcelona Health Survey)
	Fatigue	Brief Fatigue Inventory
**Breast cancer data**		
	Diagnosis and treatment	Ad hoc
**Lifestyle**		
	Tabacco	ESB-2011 (adaptation) and ad-hoc
	Alcohol	EDADES-2011 (housing survey about alcohol and drug use in Spain)
	Nutrition	Ad hoc
	Physical activity	ESB-2011 (Barcelona Health Survey) and International Physical Antivity Questionnaire - Brief
**Emotional well-being**		
	Quality of life	Two of the questionnaires from the European Organisation for Research and Treatment (EORTC) Quality of Life Questionnaire: QLQ-C30 (quality of life questionnaire in persons with cancer), QLQ-BR23 (specific for women with breast cancer)
	Mental health	Hospital Anxiety and Depression Scale, ad hoc
	Social support	Berkman-Syme Social Network Index, Modified Social Support Survey, ad hoc
**Health care services**		
	Satisfaction	Ad hoc, Australian survey from Raupach and Hiller [19], some questions from Princess Margaret Hospital Patient Satisfaction Questionnary with Medical Doctor [20], article by Grunfeld et al [21].
	Use of services	ESCA-2011 (Catalonia Health Survey)
**Unconventional therapies**		
	General therapies	Complementary and Alternative Medicine adaptation, ad hoc
	Cannabis	Ad hoc
**Sociodemographic**		
	Social	ESB-2011 (Barcelona Health Survey), ESCA-2011 (Catalonia Health Survey), 2010/11 Survey of Health Ageing and Retirement in Europe - Wave 4
	Personal	Ad hoc

#### Independent Variables

We will study the variables for objectives 1 and 2 as dependent variables, and will also evaluate whether they are related to disease progression as independent variables (dietary habits, BMI, tobacco, alcohol consumption, quality of life, social networks, emotional support, unconventional therapy use). Similarly, the dependent variables in objective 3 (TNM, stage, type of tumor, and treatment) will also be used later as independent variables. Other variables include social class, based on the woman’s job or that of the main person she lives with (according to the Spanish adaptation of the National Job Classification, CNO-2011 of the British Register General Classification), and level of education. We will also use contextual variables to perform multilevel analyses based on available family income, which is an index composed of: (1) academic qualification of the population measured as the proportion of higher degrees, (2) employment situation as the ratio of unemployed to working-age people, (3) ratio of cars to residents, (4) the power rating of new cars acquired by residents, and (5) second-hand residential market prices. Other contextual indicators can be selected according to the study variable for the various objectives (eg, distribution of green spaces, services, public transportation).

### Analysis

First, we will perform a descriptive analysis of each study variable. To test for differences in lifestyle before and after breast cancer diagnosis, we will use the appropriate paired data hypothesis tests for each data distribution, which we will stratify by socioeconomic status and cultural origin. We will perform a bivariate analysis to measure the degree of relationship between different study components within emotional well-being and unconventional therapy use according to socioeconomic level, age group, and years since diagnosis. We will compare means or percentages using student’s t-tests or analyses of variances for continuous variables, and Chi-square tests for categorical variables. We will then carry out a multilevel analysis, adjusting regression models to observe the possible relationships between the dependent variable and lifestyles, emotional well-being, use of unconventional therapy, and social class.

We will use Cox regression to perform survival analyses of the number of relapses or metastasis and death, and with the following dependent variables: lifestyle components, emotional well-being, unconventional therapy, and socioeconomic status. We will calculate the hazard ratio and 95% CI for breast cancer, controlling for covariates using the Cox model and controlling for various risks. Women who did not present any of the events of interest at the time of the analysis will be considered right-censored. We will carry out this analysis using information from successive surveys or as mortality information becomes available. In these models, we will also perform a multilevel analysis which, in addition to analyzing individual variables, takes into account a second level consisting of the relationship of the event of interest with the contextual variables mentioned above. All analyses will be performed using STATA v11 and SPSS v18.

### Profile of Women Recruited to the DAMA Cohort

**Figure 2:**
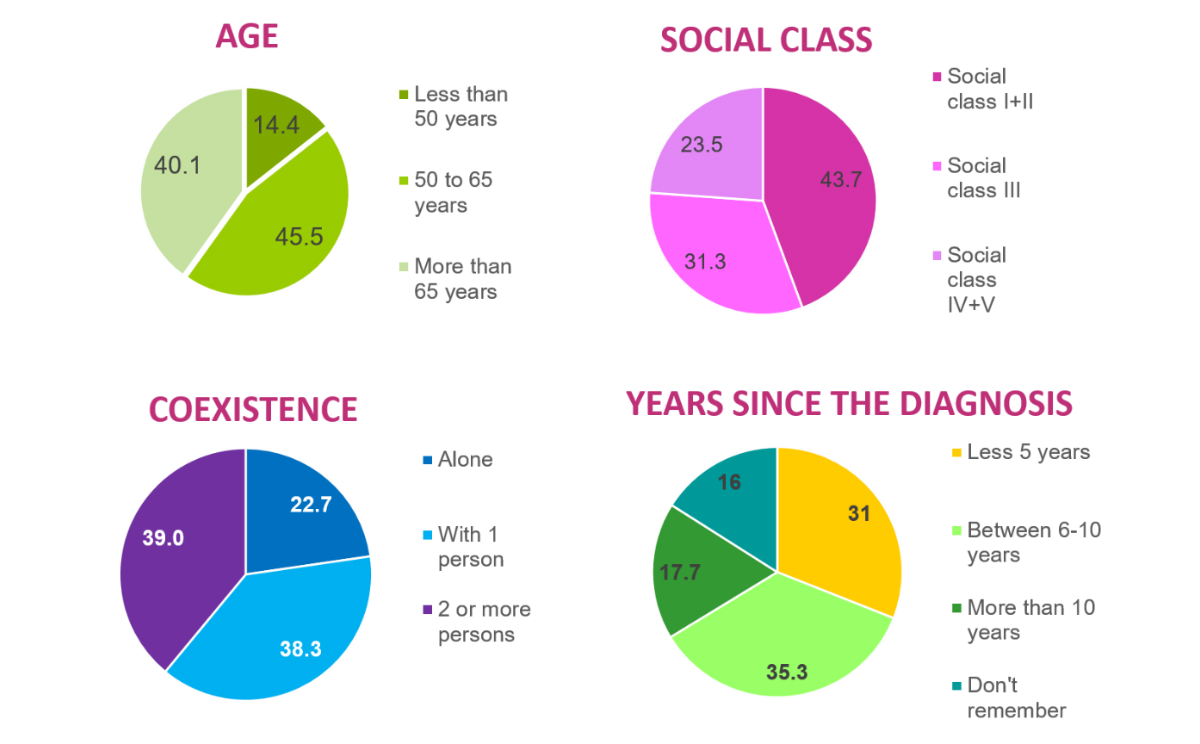
Profile of the women of the DAMA Cohort by age, social class, number of people cohabiting, and number of years since diagnosis.

We determined the final cohort based on the answers to the welcome survey, which was completed by 2712 of the women who were sent an informed consent form 98.26% (2712/2760). Forty-eight women did not respond for various reasons, but primarily because we could not locate them (Figure 1). As shown in Figure 2, 14.1% (391/2712) respondents were under 50 years of age, 45.50% (1234/2712) were between 50 and 65 years of age, and 40.08% (1087/2712) were over 65 years of age. By social class, 43.69% (1185/2172) belonged to the highest social classes (I and II), 31.27% (848/2712) to the middle class (III), and 23.49% (637/2712) to the working classes (IV and V). Forty-two respondents had missing data. In terms of cohabitation, 22.71% (616/2712) lived alone, 38.31% (1039/2712) lived with one person, and 38.97% (1057/2712) lived with two or more people.

Regarding employment status, 34.96% (948/2712) of respondents were currently working, 20.46% (555/2712) were not currently working (either on leave or for a similar reason), and the remainder were either retired 33.52% (909/2712), or had a temporary or permanent disability 8.85% (240/2712) Sixty respondents had missing data. In terms of caregiving, 14.20% (294/2070) of cohabiting women lived with someone with a disability; of these, 24.83% (73/294) took care of the disabled person themselves, 7.82% (23/294) had help from their partners, and 5.78% (17/294) had hired help. We observed various domestic work situations, the most common being the woman assuming the work herself 37.39% (1014/2712), followed by cases where the woman had hired help 28.98% (786/2712). Regarding the evolution of breast cancer, 10.99% (298/2712) of the women reported having had a local or metastatic relapse (Table 2).

Given the option of responding to the questionnaire on paper or online, 29.98% (813/2712) of respondents chose the online version. Upon receiving the completed questionnaires, we sent the women a brochure with a complete menu of healthy food options, designed specifically for the women of the DAMA Cohort by a prestigious, internationally-known chef. A total of 2235 women completed this study and the entire process lasted 15 months.

**Table 2:** Description of the sample of women that comprise the DAMA Cohort. Some data are missing due to nonresponse.

Parameter		Number	Percentage (%)
**Educational level, N=2712**			
	Primary or less	821	30.27
	Secondary	813	29.98
	University	879	32.41
	Other situations	199	7.34
**Working status, N=2712**			
	Currently working	948	34.95
	Not currently working	555	20.46
	Disability	240	8.84
	Retired	904	33.33
**Living with a disabled person, N=2070**			
	Yes	294	14.20
**Who cares for this disabled person? N=294**			
	The subject alone	73	24.83
	Shared with the partner	23	7.82
	Shared with a hired helper	17	5.78
**Who performs domestic tasks? N=294**			
	The subject alone	1014	37.36
	Shared with the partner	418	15.41
	Shared with a hired helper	786	28.98
**Relapse, N=2712**			
	Yes	298	10.98
	No	2392	88.20

## Discussion

This is the first study performed in our context that is focused on analyzing different aspects that concern women diagnosed with breast cancer, but still remain unknown among health care professionals. The lack of knowledge, in combination with higher levels of patient interest over time, may have allowed us to successfully create the DAMA cohort with high levels of participation. Considering the total study population of 10,612 women, the theoretical sample size was 1928, which was markedly overcome by the actual number of participants enrolled in the DAMA cohort.

### Limitations and Strengths

This study presents a number of limitations but also has strengths and important contributions. The fact that this is a self-administered survey with two ways to respond (online and on paper) introduces an initial bias since the choice depends on the type of woman answering the questionnaire. Furthermore, the online survey makes many questions obligatory in order to proceed, such that the paper survey has a higher probability of blank or poorly answered responses.

There is an implied and inevitable memory bias due to the fact that participants are asked about events that occurred a long time ago (in some cases more than 10 years earlier) and often before their breast cancer diagnosis. The same bias is also triggered by the fact that, as one of the participants mentioned, some women were reminded of issues they wanted to forget, in contrast to other women who had experienced them recently. This problem implies that the bias will operate in opposing directions.

Despite being the most important hospitals in Barcelona, which care for the largest number of women (approximately 85% of the total number of cases of breast cancer), the fact that we created this cohort using only patients that were in public hospitals implies that we probably do not have a representative sample of all women with breast cancer. In addition, more than 10,000 women were invited to participate, but those who accepted voluntarily participated. Therefore, a volunteer bias also exists.

It would be desirable to compare the participants of the study with the nonparticipants. However, the information available comes from hospital records, due to the lack of an official Cancer Registry, which do not allow this comparison. We still know that, in terms of age, the women who make up the DAMA Cohort are representative of the total number of women with breast cancer in Catalonia. Nonetheless, this is the first time that a study with these characteristics has been carried out in our setting, and that explores factors beyond the hospital environment (that are also very important) that often leave women with a feeling of helplessness. This issue implies that women’s personal resources and their social and structural environment are important for their opportunities and capacity to find day-to-day solutions to face the new personal challenges that arise in their new circumstances of dealing with the disease.

Perhaps for this reason, participation in the study was higher than expected, which is one of its strengths. The Welcome Survey was also key in raising women’s awareness of being part of the cohort, as a way to establish direct and personalized contact with them.

The fact that this study deals with such diverse issues will allow us to solve hypotheses and answer questions while raising new ones. Over time we will be able to establish causal relationships, but for now this study already allows us to make some estimations because it includes women who have had the disease for different lengths of time, and at various stages of progression.

The information obtained will allow us to identify the main shortcomings, needs, and difficulties that women face after treatments for breast cancer. The study will also identify which population groups are more vulnerable than others; from this information we will be able to establish priorities upon which to intervene. This information will lay the foundation for designing and evaluating interventions that, if effective, can be integrated into health services and be equitably offered to all women with breast cancer.

## Abbreviations

BMI: body mass index
CAM: complementary and alternative medicine
DAMA: Dones Amb Càncer de Mama
FEDER: Fondos European Regional Development Fund
TNM: tumor nodes metastases
